# Severe early ovarian hyperstimulation syndrome following GnRH agonist trigger and freeze-all strategy in GnRH antagonist protocol; case report and literature review

**DOI:** 10.5935/1518-0557.20220065

**Published:** 2023

**Authors:** Nazanin Hajizadeh, Sedigheh Hosseini, Saghar Salehpour, Hajar Abbasi, Jehan Saheb

**Affiliations:** 1 Preventative Gynecology Research Center, Shahid Beheshti University of Medical Sciences, Tehran, Iran

**Keywords:** GnRH agonist triggering, GnRH antagonist, freeze-all, ovarian hyperstimulation syndrome

## Abstract

Ovarian hyperstimulation syndrome (OHSS) is characterized by increased vascular permeability, hemoconcentration and fluid leakage to the third space. The vast majority of OHSS cases occur following ovarian stimulation for IVF. This potentially lethal iatrogenic condition is one of the most serious complications of assisted reproductive technologies. We report one case of severe early OHSS after GnRH agonist trigger in a GnRH antagonist protocol and freeze-all approach without the administration of any hCG for luteal-phase support in a 34-year-old case of PCO with 7 years primary infertility. After oocyte retrieval the patient was seen at the emergency unit of the hospital with abdominal distension, pain, anuria, dyspnea, and OHSS symptoms. The diagnosis was OHSS with severe ascitis. She was admitted to the Intensive care unit (ICU). She was managed with oxygen by mask, intravenous fluids, anticoagulant and albumen, we performed a two-time vaginal ascites puncture, resulting in the removal of 7800mL of clear fluid in Intensive Care Unit with full recovery. This case study presents the clinical manifestations, investigation, progress, management, outcome and preventive measures. The patient was managed with no complications. Clinicians have to be aware that even the sequential approach to ovarian stimulation with a freeze-all approach and GNRH analog triggering does not completely eliminate OHSS in all patients.

## INTRODUCTION

Ovarian hyperstimulation syndrome (OHSS) is the most severe and potentially life-threatening iatrogenic complication associated with ovulation induction. OHSS is an exaggerated response to COS characterized by the shift of protein-rich fluid from the intravascular space to the third space due to increased vascular permeability in response to stimulation by either exogenous or endogenous HCG (Rastin *et al*., 2019). Vascular endothelial growth factor (VEGF) has been identified as the major mediator. Prostaglandins, inhibin, the renin-angiotensin-aldosterone system, and inflammatory mediators have all been implicated in OHSS etiology. The incidence of OHSS is varied; 33% of IVF cycles have been reported to be associated with mild OHSS; whereas the more severe forms have been reported in 2%-6% of IVF cycles (Nakatsuka *et al*., 2019; Caretto *et al*., 2017).

Having followed the above-mentioned strategy for high-risk OHSS patients, we hereby present one case in which the segmentation approach resulted in the development of severe OHSS, requiring hospitalization and peritoneal drainage.

## CASE REPORT

A 34-year-old woman from Tehran in IRAN with 7-year primary infertility, was referred to the fertility center. She had irregular menstrual cycle with oligomenorrhea and moderate to severe hirsutism. Her body mass index was 31.1 kg/m^2^ (body weight, 73 kg). The basal ultrasound showed normal uterus, polycystic ovaries and more than a total of 25 antral follicles; normal patency of both tubes and normal uterine cavity were seen in her hysterosalpingogram. The sperm count of the partner yielded asthenoteratospermia.

### Ovarian stimulation protocol

The patient was stimulated in a flexible GnRH antagonist protocol. Stimulation with recombinant FSH(Cinnal-f) 150 IU and HMG (Pd, Homog)75 IU started on day-2 of the menstrual cycle, and the GnRH antagonist was administered from day-6 of stimulation (cetrotide). The stimulation lasted for 9 days. The ultrasound examination showed seven follicles of 17 mm in diameter, 16 follicles of 15 mm in diameter, and >10 follicles between 10 and 14 mm in diameter in both ovaries. Final follicular maturation was induced with a bolus of GnRHa (Variopeptyle, 0.3 mg). A total of 21 oocytes were retrieved, resulting in the vitrification of 13 day-3 embryo (A and A- B quality). Because of the high number of follicles present at oocyte pickup, the dopamine agonist (cabergoline 0.5 mg) and GnRH antagonist (cetrotide 0.25mg) were also administered after oocyte pickup on a daily basis.

### Follow up

Day-6, after oocyte retrieval, the patient was seen at the emergency unit of the hospital with abdominal distension, pain, anuria, dyspnea, and OHSS symptoms. A blood count revealed: hematocrit 39%, hemoglobin 14.2 g/dl, platelet count of 418,000, white blood cell 20000 µL, Cr 2.1 mg/dl and Alb 2.3 g/dL. The ultrasound scan revealed severe enlarged ovaries of 14cm in diameter each and severe ascites.

The weight of the patient increased by 3kg (76 kg). Her coagulation profile remained normal. She was treated; half saline infusion 500 CC stat and then, based on the urine output, continuation of dopamine agonist (cabergoline 0.5 mg daily), GnRH antagonist (cetrotide 0.25mg daily) and low molecular heparin (enoxaparin, 40 mg) and antithrombosis. We performed a vaginal ascites puncture, resulting in the removal of 5,300 mL of clear fluid. Before, during, and 2 hours after the ascites puncture, we administered 2,400 mL of saline solution, as well as 100 mL of albumin. The urine output during the following days varied between 450 and 550 mL.

At the second day of admission, the patient had oliguria and increased abdominal distention. We then performed vaginal ascites puncture, resulting in the removal of 2,500 mL clear fluid. After drainage and IV infusion, the hematocrit decreased to 36.1%; WBC to 15,120; platelet count to 381,000, and hemoglobin (Hgb) of 13.7 g/dL. Three days later the patient was discharged and followed up in an outpatient clinic. Menses occurred as late as 14 days after the oocyte retrieval.

## DISCUSSION

Human chorionic gonadotropin has been the gold standard for final oocyte maturation. HCG and LH bind to and activate the same receptor, but the half-life of LH is 60 minutes (Thakre & Homburg, 2019), whereas that of HCG is 24 hours (Evbuomwan, 2013). Hence, HCG exerts sustained luteotropic activity and may induce the occurrence of OHSS.

The GnRHa-induced surge effectively stimulates ovulation and oocyte maturation, but differences exist regarding the duration and profile of the GnRHa-induced surge of gonadotropins when compared with that of the natural cycle (Lazzaretti *et al*., 2019). Thus, the GnRHa-induced surge lasts for approximately 24 hours, whereas in the natural cycle the endogenous LH surge lasts approximately 48 hours, leading to a significant reduction in the total amount of gonadotropins released during the GnRHa-induced surge.

The shorter duration of the endogenous LH surge, induced by GnRHa triggering as compared with the continuous LH/hCG receptor stimulation for an estimate of 7-9 days with a bolus of 10,000 IU hCG, in combination with a negative impact of the supraphysiologic steroid level of LH secretion by the pituitary after COS, with a complete change in LH secretion pattern after agonist trigger, are the most plausible explanation behind the reduced risk of OHSS when GnRHa is used to trigger final oocyte maturation.

To date there has been only one report of allegedly severe early-onset OHSS after GnRHa trigger in the English literature (Montanelli *et al*., 2004). There has been one retrospective report of a high incidence of early-onset OHSS (22%) after GnRHa trigger, but in a population of high-responder patients who received a low-dose hCG rescue protocol despite the retrieval of between 50 and 65 metaphase II oocytes in some patients (Di Carlo *et al*., 2012). In contrast, in a prospective pilot study (n = 12) in high-responder patients, there was one case of late moderate OHSS that did not require hospitalization (Sonntag & Keck, 2019). The largest randomized, controlled trial published to date in an OHSS high-risk population consisting of patients with a follicle count between 15 and 25 follicles (Du *et al*., 2020), did not show any OHSS development, despite the use of the aforementioned low-dose hCG rescue protocol followed by a fresh embryo transfer. Importantly, the reproductive outcome was excellent and similar to that of the HCG trigger.

Sporadic and familial cases of spontaneous OHSS have generated an interest in genetic mechanisms for OHSS development independent of exogenous gonadotropins. Genetic studies of OHSS have focused on the FSH receptor (FSHR) gene and its mutations, as well as targeting VEGF receptors as a method to modulate OHSS. Activating mutations in the FSHR contributes to OHSS; whereas inactivating it causes sterility. During the last decade, several cases of spontaneous and familial OHSS have been reported, suggesting a possible genetic cause for OHSS. The discovery of activating FSHR mutations that turn the receptor sensitive to stimulation by HCG (Darii *et al*., 2019).

In conclusion, A number of strategies have been used to reduce the risk of OHSS, such as the use of a GnRH antagonist protocol with low gonadotropin doses and GnRH agonist trigger, coasting, cycle cancellation, freeze-all strategy and administration of dopamine agonist and GNRH antagonist. We report one case of severe early OHSS after GnRH agonist trigger in a GnRH antagonist protocol and freeze-all approach. According to article reviews, GnRH receptor, FSHR, or LH receptor gene mutations is possible for OHSS predisposition. More studies and research are needed to investigate a clinically useful connection between the receptor mutations and the occurrence and intensity of the OHSS. Hence, the antagonist protocol with GnRHa trigger and freeze-all approach does not completely eliminate OHSS in high-risk patients.
